# Lipidomic Characterization of Marine By-Product Oils: Impact of Species and Extraction Methods on Lipid Profile and Antioxidant Potential

**DOI:** 10.3390/antiox15010095

**Published:** 2026-01-12

**Authors:** Ioannis C. Martakos, Paraskeui Tzika, Marilena E. Dasenaki, Eleni P. Kalogianni, Nikolaos S. Thomaidis

**Affiliations:** 1 Laboratory of Analytical Chemistry, Department of Chemistry, National and Kapodistrian University of Athens, Panepistimiopolis Zographou, 15772 Athens, Greece; johnmrtk@chem.uoa.gr; 2 Department of Food Science and Technology, International Hellenic University, 57400 Thessaloniki, Greece; tzikap@food.ihu.gr (P.T.); elekalo@ihu.gr (E.P.K.); 3 Laboratory of Food Chemistry, Department of Chemistry, National and Kapodistrian University of Athens, Panepistimiopolis Zographou, 15772 Athens, Greece; mdasenaki@chem.uoa.gr

**Keywords:** marine oils, marine by-products, lipidomics, tocopherols, carotenoids, extraction methods

## Abstract

Marine by-products represent an important source of bioactive lipids with potential applications in nutraceuticals and functional foods. This study provides a biochemical and lipidomic characterization of oils derived from sardine, monkfish, grey mullet roe, squid, and anchovy by-products, assessing how the extraction method influences their lipid and antioxidant profiles. Fatty acids were quantified by GC-FID, antioxidant compounds by HPLC-DAD, and untargeted lipidomics by TIMS-HRMS. A total of 228 lipid species were identified, predominantly triglycerides (TGs) and diglycerides (DGs), accounting for approximately 69% of the annotated lipidome. Grey mullet roe oils exhibited the highest levels of long-chain PUFAs (EPA, DHA) and antioxidants (α-tocopherol 205–469 mg/Kg, lutein 10–125 mg/Kg, and squalene 1004–6049 mg/Kg), whereas squid oils showed high n-3/n-6 proportions. The extraction method strongly affected lipid integrity. Supercritical CO_2_ extraction with ethanol (SFE–SE) preserved the greatest proportion of PUFA-rich TGs, yielding ~27–28 g EPA + DHA per 100 g oil, while wet reduction and mechanical pressing produced lower PUFA levels (~22 g/100 g) and increased hydrolysis/oxidation-associated lipids. PCA and PLS-DA revealed clear clustering driven by species and extraction class, with PUFA-containing TGs and DGs identified as major discriminating lipids. These results highlight the critical role of extraction conditions in determining the nutritional and functional value of marine oils and support the valorization of marine by-products in high-value applications.

## 1. Introduction

Marine by-products such as heads, viscera, skin, and roes represent a sustainable and under-utilized source of bioactive lipids and antioxidants [1,2]. Marine-derived oils are rich in omega-3 fatty acids and represent an important opportunity for valorization due to their high content of bioactive lipids, including polyunsaturated fatty acids (PUFAs) such as eicosapentaenoic acid (EPA), docosahexaenoic acid (DHA), and monounsaturated fatty acids (MUFAs), which contribute to improved metabolic health and enhanced cognitive function, while they have strong antioxidant, anti-inflammatory, and cardiovascular-protective properties, making them valuable for nutraceutical, pharmaceutical, and cosmetic applications [3,4,5,6]. In addition, marine-derived fish oils contain high concentrations of a wide range of common lipid classes and subclasses, including phospholipids, triglycerides, diglycerides, and sterols, compounds that have demonstrated superior bioavailability [7].

Additionally, marine organisms are a great source of important antioxidant compounds, which play a crucial role against oxidative stress caused by environmental factors, enhance the immune function, improve survival rates, and protect the cells by scavenging free radicals and reducing lipid peroxidation [8,9,10,11]. These compounds include carotenoids, tocopherols, and squalene, and they also contribute by enhancing the nutritional value of these organisms [9]. Beyond their role in enhancing the nutritional value of marine organisms, these antioxidants are key to the preservation and stability of seafood products [10]. Specifically, carotenoids and tocopherols have been linked to scavenging free radicals, reducing lipid peroxidation while also ensuring nutritional quality, and squalene is linked to reactive oxygen species (ROS) neutralization, with several studies in the literature focusing on the antioxidant and cell-protective nature of these compounds [10,12,13]. Consequently, the substantial bioactivity of these compounds establishes their significant value across pharmaceutical, nutraceutical, and skincare applications.

Beyond traditional dietary supplementation, marine bioactive lipids have gained prominence in the nutraceutical and cosmetic industries. The nutraceutical sector incorporates omega-3-rich oils into functional foods, supplements, and pharmaceutical formulations aimed at preventing chronic diseases [14]. Marine-derived lipids are increasingly used in cosmeceuticals for their ability to reinforce skin barriers, enhance hydration, and reduce oxidative stress-induced skin aging [15,16]. Omega-3 and omega-6 fatty acids help regulate inflammatory skin conditions such as eczema, psoriasis, and acne. Studies have shown that incorporating marine phospholipids into topical skincare formulations can lead to improved skin elasticity, moisture retention, and UV-damage protection [17].

The efficacy of these bioactive lipids depends largely on their stability and preservation during extraction. As marine lipids are highly susceptible to oxidation and degradation, selecting an appropriate extraction method is crucial for maintaining their bioactivity and functional properties. Furthermore, different methods and solvents result in the extraction of lipids of different polarities and, therefore, affect the bioactive content of the oils [18,19,20]. Industrial-scale oil from fish by-products is extracted mainly via “wet reduction”, where the by-products undergo hydrothermal treatment, followed by separation methods to obtain the oil (pressing and/or centrifugation and/or decantation and finally drying) [21]. However, other methods, including supercritical CO_2_ (SFE-CO_2_) extraction, enzymatically assisted hydrolysis, and solvent extraction methods, have been studied to optimize yield while preserving the structural integrity of omega-3 fatty acids, phospholipids, and antioxidants. Furthermore, in recent years, methods considered more sustainable, such as SFE-CO_2_, ultrasound-assisted extraction, microwave-assisted extraction, and enzymatically assisted hydrolysis, have been highlighted within the literature [19,22,23]. Studies comparing SFE-CO_2_ extraction (SFE) with conventional solvent-based methods indicate that SFE better preserves lipid oxidation stability, significantly reducing peroxide values and maintaining the integrity of bioactive compounds such as tocopherols, phospholipids, and omega-3 fatty acids. Research on fish oil extraction from Arctic charr and fish roe highlights that supercritical CO_2_ prevents oxidative degradation due to the absence of oxygen and the low-temperature conditions, resulting in superior retention of polyunsaturated fatty acids (PUFAs) and antioxidants compared to solvent-based methods [24,25]. Furthermore, recent research highlights the low-temperature continuous phase-transition extraction (LCPE) method as a promising approach for maintaining oxidative stability in Antarctic krill oil, reducing lipid degradation while maximizing bioactive compound retention [26]. These findings emphasize the need to refine extraction protocols to ensure that marine-derived lipids retain their bioactivity, making them viable for long-term nutraceutical formulations and cosmetic applications.

The aim of this study is to comprehensively evaluate how different extraction methods affect the lipid composition and antioxidant content of oils obtained from various by-products of Greek fisheries. Five distinct methods were grouped and compared based on their mechanisms of action: three conventional methods (wet reduction, enzymatic hydrolysis, and mechanical pressing) are compared with newer and more sustainable ones, such as SFE and green solvent extraction (with the use of ethanol). Particular focus is placed on omega-3 and omega-6 fatty acids, along with key antioxidant compounds such as α-tocopherol, lutein, and squalene. By integrating targeted biochemical assays with untargeted lipidomic profiling, this work seeks to clarify how extraction conditions influence the nutritional and functional potential of marine oils. The inclusion of diverse by-products from commercially important marine species reflects an effort to promote full-resource utilization and contribute to more sustainable, circular approaches in seafood processing—particularly in the context of nutraceutical and cosmetic applications.

## 2. Materials and Methods

### 2.1. Chemicals and Standards

Methanol (MeOH), ethanol (EtOH), isopropyl alcohol (IPA), and acetonitrile (ACN), HPLC grade, as well as methyl-tert-butyl ether (MTBE) and hexane, analytical grade, were purchased from Fisher Scientific (Geel, Belgium). ACN and IPA of LC-MS grade were obtained by Merck (Darmstadt, Germany). Ammonium formate, formic acid, potassium hydroxide, and sodium sulfate were acquired by Sigma-Aldrich (Steinheim, Germany). A Milli-Q water purification system (Direct–Q, UV, Millipore, Bedford, MA, USA) was used to provide ultra-pure water (18.2 MΩ/cm). Standard compounds, including lutein, β-carotene, α-tocopherol, γ-tocopherol, δ-tocopherol, and squalene, were obtained from Sigma Aldrich. FAME mix containing a 37-component mix was supplied by Supelco. Chromafil 0.22 μm regenerated cellulose (RC) filters were acquired by Macherey-Nagel (Düren, Germany).

### 2.2. Samples and Extraction Techniques

In total, 19 samples of marine oils were analyzed in the Laboratory of Analytical Chemistry of NKUA. The marine oil samples originated from 6 different marine species by-products, namely sardine heads (*Sardina pilchardus*), monkfish bellies (*Lophius piscatorius*), grey mullet roes (*Mugil cephalus*), squid bellies (*Nototodarus sloanii*), and anchovy heads (*Engraulis encrasicolus*). All fish were caught in the FAO 37.3.1 region, whereas the squid was caught in the FAO 81 region and processed by Atlantida S.A. A total of 5 extraction methods were used and assessed: wet reduction (WR), mechanical pressing (MP), solvent extraction with the use of ethanol (EtOH) (SE), enzymatically assisted hydrolysis extraction (EH), and supercritical fluid extraction with CO_2_ and ethanol as a cosolvent (SFE-SE). All samples were extracted on the premises of the International Hellenic University (IHU).

Extraction methods (WR, MP, SE, and SFE-SE) were applied as described previously [18,19,20]. Briefly, WR was applied in a mixture of finely cut raw mullet roe by-product (300 g) and water (75 g). The mixture was heated under stirring (water bath, 90 °C, 10 min) and then separated by passing twice in an expeller oil press (OW 500 s-inox oil press, Oelwerk, Cottbus, Germany) and subsequently centrifuged (RC3, Sorvall) at 4132× *g* for 15 min. MP was the same as WR without the hydrothermal treatment. For SE, ethanol was used as a solvent, and a ratio of 1/10 (weight of freeze-dried by-product/volume of solvent) was applied at two temperatures (25 and 50 °C) for 2 h. The oil was obtained by centrifugation (1878× *g* for 15 min, Rotofix 32A, Hettich, Tuttlingen, Germany) and vacuum evaporation (R-210 Rotavapor, Buchi) at 37 °C. Finally, EH was applied as in Kalogianni et al. [27]. Briefly, first the endogenous enzymes were inactivated (90 °C for 10 min), then the sample was cooled to 55 °C, and 1.0% (*w*/*w*) of Alcalase ^®^ or 0.5% (*w*/*w*) Protease ^®^ was added and incubated at the same temperature for 1 h. Then, the added enzymes were inactivated as above, and the oil was recovered by centrifugation, as above. The mixture was incubated in a water bath at 55 °C with continuous stirring, and samples were taken at 35 min, 1 h, 2 h, and 3 h. For all samples, the added enzymes were inactivated at 90 °C for 10 min, and then the system was cooled at 4 °C. Finally, the oil was recovered using centrifugation with an RC3 Sorvall (Thermo Scientific, Waltham, MA, USA) centrifuge, operating at 4 °C at a force of 4132× *g* for 15 min [19]. Conventional extraction techniques were considered: wet reduction, enzymatically assisted hydrolysis, and mechanical pressing. These techniques offer a sustainable alternative, minimizing chemical exposure while preserving the natural structure of marine bioactive lipids.

By categorizing the samples into these three groups, this study enables a systematic comparison of lipid and antioxidant compound retention across different extraction methodologies. The classification allows for a better understanding of the effects of each technique on the final lipid profile, contributing to the optimization of extraction processes for nutraceutical and cosmetic applications. In Table S1 of the Electronic Supplementary Material (ESM), the samples alongside their detailed information are presented.

### 2.3. Determination of FAME and Antioxidant Profile

#### 2.3.1. FAME Analysis

For the determination of FAMEs, the method 996.06 of AOAC was used. Briefly, 0.050 g of fish oil was accurately weighed in an Eppendorf tube, followed by the addition of 750 μL of hexane. The sample was vortexed to ensure proper mixing, and 100 μL of 2 M KOH/MeOH was added. After vortexing for 1 min, 0.2 g of Na_2_SO_4_ was added, and the mixture was mixed vigorously. The sample was subsequently frozen at −20 °C for 30 min. After freezing, 100 μL of the supernatant was collected and diluted with 900 μL of hexane. Finally, 1 μL of the prepared sample was injected into the GC-FID system for further analysis. The GC system comprised a Varian 450 GC with a Flame Ionization Detector (FID) with the settings presented in Table 1.

**Table 1:** GC-FID system parameters and settings.

Analytical Column	Agilent J&W DB-23 GC (60 m, 0.25 mm)
Carrier Gas	Helium
Injection System Temperature	250 °C
Detector Temperature	280 °C
Oven Temperature Program	Initial 50 °C for 1 min, ramping at 25 °C/min to 175 °C, then ramping at 4 °C/min to 230 °C, holding for 10 min
Hydrogen Flow Rate	40 mL/min
Synthetic Air Flow Rate	450 mL/min
Helium Flow Rate	30 mL/min
Split Ratio	1:50
Injection Volume	1 μL

#### 2.3.2. Tocopherols, Carotenoids, and Squalene Analysis

For the determination of tocopherols, carotenoids, and squalene, a method previously published [28] was used. Briefly, 100 μL of oil sample was weighted in an eppendorf tube, followed by the addition of 900 μL of IPA. The sample was mixed vigorously with the use of a vortex mixer and was filtered by a 0.22 μm RC filter. Finally, 20 μL were injected in the HPLC system. For this analysis, a Shimadzu 2030AD HPLC system was used. The system consisted of an autosampler, a column thermostat, and a photo-diode array detector (PDA). Samples were measured in duplicates. The identification and detailed characterization of the target compounds, including retention time and chemical structure, were performed as described in Martakos et al. (2019) [28].

### 2.4. Lipidomics Analysis

For the lipidomic analysis, 100 mg of the sample was weighed into an Eppendorf tube, followed by the addition of 600 μL of an MTBE:MeOH (3:1) extraction system. The sample underwent vigorous vortex mixing and was then placed in an ultrasonic bath for 30 min at 30 °C. After sonication, 500 μL of water was added, and the sample was centrifuged at 4 °C to separate the phases. The upper MTBE layer, containing the extracted lipids, was collected, and a second extraction was performed to ensure complete lipid recovery. The two MTBE layers were then combined, and the solvent was evaporated under a gentle nitrogen stream to dryness, at room temperature. The dried lipid extract was subsequently reconstituted in 200 μL of ACN:IPA (1:1) before injection into the LC-MS system.

For the comprehensive analysis of the lipid profile of fish oils, ultra-performance liquid chromatography coupled with trapped ion mobility spectrometry and high-resolution quadrupole time-of-flight mass spectrometry (UPLC-TIMS-QTOF-MS) was utilized. The chromatographic separation was performed using a Bruker Elute UPLC system equipped with a Thermo Acclaim RSLC 120 C18 analytical column (2.2 μm, 2.1 × 100 mm) and an Acquity UPLC BEH C18 VanGuardTM pre-column (2.1 × 5 mm, 1.7 μm; Waters). The mobile phase consisted of ACN:H_2_O (65:35) as phase A and ACN:IPA (15:85) with 10 mM ammonium formate and 0.1% formic acid as phase B. A gradient elution was applied, starting at 30% B, gradually increasing to 100% B over 30 min, at a constant flow rate of 0.25 mL/min. Detection was performed using a Bruker TIMS-TOF PRO 2 mass spectrometer, enabling high-resolution analysis of lipid species by adding the 4th dimension of ion mobility. The TIMS-TOF instrument was operated using the parameters described in Martakos et al. (2024) [29].

Due to the complexity of LC–MS datasets and the extensive processing required [30], specialized software was used. MetaboScape 2023b and DataAnalysis 5.3 (Bruker Daltonics, Bremen, Germany) were employed for spectral processing, feature extraction, and compound annotation. Metaboscape 2023b was utilized for peak-picking, lipid metabolite annotation, and lipid identity confirmation, with additional verification performed using Data Analysis 5.3. Peak-picking was conducted using the T-ReX 4D algorithm, which integrates four key parameters—retention time, signal intensity, mass-to-charge ratio (*m*/*z*), and collision cross-section (CCS) value—to enhance metabolite detection accuracy. To minimize false positives, only features detected in at least 50% of samples were considered. For lipid annotation, Metaboscape’s Lipid Annotation Tool was employed alongside the Lipidblast spectral library [31], applying stringent criteria: an *m*/*z* tolerance of 5.0 ppm, an isotope fit threshold of 250 mSigma, MS/MS matching scores up to 400/1000, and a CCS value tolerance within 3.0%. Features exceeding these thresholds were excluded to reduce false positives, particularly since Lipidblast incorporates in silico-generated CCS values, requiring careful validation. Batch correction was applied in Metaboscape using quality control (QC) samples to account for systematic variations, with variance thresholds set at 40% before correction and 20% post-correction. Features that exceeded these thresholds or were absent in QC samples were excluded from further analysis.

Statistical analyses were carried out using MetaboAnalyst 6.0 [32], incorporating Principal Component Analysis (PCA) and Partial Least Squares Discriminant Analysis (PLS-DA), with violin and box plots used for data visualization. Probabilistic Quotient Normalization (PQN) was applied to correct for sample-to-sample variability and ensure comparability across samples, a standard approach for metabolomic datasets lacking chemical standards [33].

## 3. Results and Discussion

### 3.1. Characterization of Marine By-Product Oils: Lipid and Antioxidant Composition

A broad array of lipid and lipid-soluble molecules was identified in the samples, including omega-3 and omega-6 fatty acids, phospholipids, triglycerides, diglycerides, tocopherols, carotenoids, and squalene.

**Figure 1:**
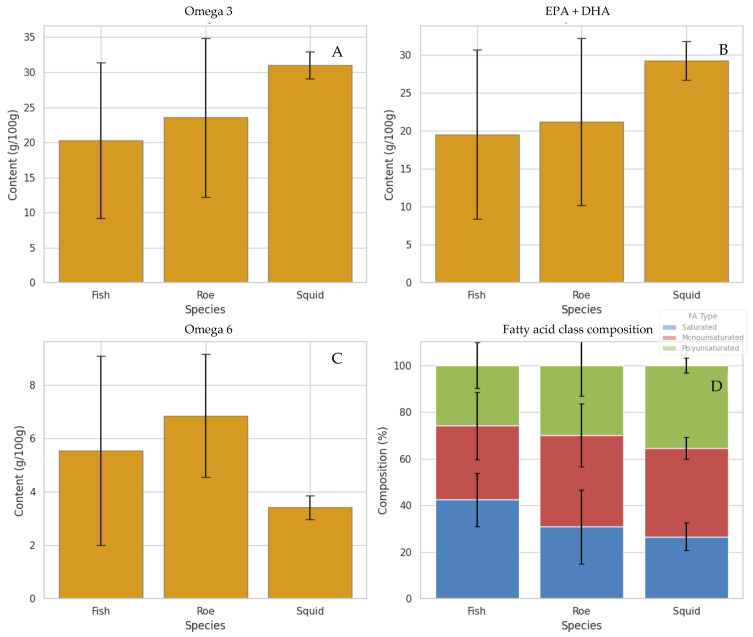
Average fatty acid composition across the marine species analyzed, presented as Mean ± Standard Deviation. “Fish” refers to the group of fish by-product samples listed in the ESM (Table S1), “Roe” corresponds specifically to grey mullet roe samples, and “Squid” refers to squid by-product samples (bellies). Values represent the mean of all samples within each species group. (**A**) Total omega-3 content (%), (**B**) sum of EPA and DHA (%), (**C**) total omega-6 (%), and (**D**) fatty acid class composition (%).

As expected, the different marine species and extraction techniques presented differences in the fatty acid composition. In Figure 1, the average content of omega-3, omega-6, EPA + DHA, and fatty acid class (saturated, MUFAs, and PUFAs) per marine species is presented. The Figure illustrates clear differences in fatty acid profiles between species, highlighting the diversity in their nutritional quality. Notably, squid bellies and grey mullet roe clearly stand out among the species by-products for their higher average levels of total omega-3 and combined EPA + DHA, as illustrated in Figure 1. Squid, in particular, exhibits the highest omega-3 and EPA + DHA content, followed by roe, while fish display comparatively lower values. These findings suggest that oils derived from squid and roe may offer superior nutritional and health benefits, given the well-established roles of long-chain polyunsaturated fatty acids in supporting cardiovascular, cognitive, and anti-inflammatory functions. Furthermore, the fatty acid class composition highlights that squid and roe oils have a more favourable balance of polyunsaturated to saturated fatty acids compared to fish, reinforcing their potential as high-quality sources of marine lipids [5,34]. The results on mullet roe by-products are in agreement with our previous works [18,19].

Conversely, fish samples exhibit notably lower average omega-3 content than roe and squid, which may limit their value as dietary sources of these essential fatty acids. A similar pattern emerges with omega-6 levels—roe samples tend to have the highest omega-6 concentrations, which could contribute to an imbalanced omega-6 to omega-3 ratio, a factor often associated with pro-inflammatory states and adverse health outcomes [35]. When examining the overall fatty acid composition by species, fish oils appear relatively richer in saturated and monounsaturated fats, while roe and squid demonstrate higher proportions of polyunsaturated fats, particularly omega-3. However, it is important to note that proportional omega-3 or PUFA levels do not fully reflect the nutritional contribution of each species. Total lipid content varies substantially, and fish typically contain considerably higher overall fat levels, making them significant dietary sources of omega-3 fatty acids despite their lower proportional values [36,37]. Thus, the present comparison focuses on compositional differences rather than nutritional adequacy, as each species contributes to omega-3 intake in different ways.

The present study evaluated the concentrations of three key bioactive lipophilic compounds—α-tocopherol, lutein, and squalene—which are recognized for their antioxidant properties and roles in supporting cardiovascular, immune, and ocular health [28]. Although limited studies are available in the literature for direct comparison, our findings are discussed in relation to previously published data to contextualize the nutritional potential of the oils analyzed. It is important to note that the composition and antioxidant content of marine oils are closely influenced by both the biological origin of the raw material and the parameters of the extraction process, including pressure and temperature [7,38]. As such, some compounds were not detected in all samples analyzed. The results are summarized in Table 2.

α-Tocopherol content ranged from 205 to 469 mg/Kg, with the highest concentration observed in sample ROE6, a sample produced by ethanol extraction at 25 °C. These levels substantially exceed those reported in unprocessed roe, such as 66.3 mg/Kg in fresh Chinook salmon roe [39] and 41 mg/Kg in avgotaracho, a traditional salted and wax-coated mullet roe product [40]. While some enriched or lipid-extracted samples, including mature roe oils, have reached up to 110 mg/100 g oil [39,41], such values pertain to the oil fraction and are not directly comparable to whole-sample measurements, as used in the present study. Lutein concentrations ranged from 10 to 125 mg/Kg, again peaking in ROE6. These values significantly exceed those previously reported for salmon roe, where concentrations between 0.24 and 0.46 mg/Kg have been documented in fresh and dried samples, respectively [42]. Although herring roe has been cited for its relatively high lutein content at 6.4 mg/g lipid, this corresponds to substantially lower levels on a whole-sample basis when typical lipid content is accounted for [41]. The elevated lutein levels observed here highlight the potential of roe oils as effective sources of dietary xanthophylls, which are highly valued for their roles in ocular function and antioxidant defence [43]. Squalene concentrations varied from 1301 to 6049 mg/Kg, with the highest value again found in ROE6. These levels greatly exceed those reported in comparable roe products such as avgotaracho (54 mg/Kg) [40] and are consistent with concentrations found in certain fish by-products, including viscera, bones, and heads, as well as in dried or salted fillets [44]. While shark liver oil remains the most concentrated known source of squalene—reportedly containing over 40% by weight [12]—its use is primarily restricted to pharmaceutical and cosmetic industries in EU when it is derived from sharks, due to sustainability and ethical concerns. The results on antioxidant species found in the present work are in alignment with the oxidative state of the oils examined previously [19]. Therefore, the appropriate choice of extraction method can provide oils rich in bioactive antioxidant species, which protect them from oxidation, as was found for mullet roe by-products [18,19], and provide valuable bioactives for humans. This is also supported by our previous findings on the in vitro biological effects of the mullet roe oils against platelet oxidation, where there seems to be a link between the concentration of antioxidants found in the present work and the in vitro antioxidant potential of the oils [20]. The high levels of squalene observed in roe oils derived from by-products in this study position these materials as nutritionally valuable and environmentally viable alternatives for dietary squalene supplementation.

The consistently elevated levels of all three bioactive compounds—particularly in grey mullet roe-derived samples—underscore the nutritional richness and functional potential of grey mullet roe oils. These findings not only reinforce the utility of roe as a dense source of health-promoting lipids but also highlight the influence of species selection, tissue maturity, and processing conditions on nutrient retention. In comparison to published data, our results identify roe oils as among the most promising natural matrices for the delivery of α-tocopherol, lutein, and squalene. Based on the samples analyzed in this study, solvent extraction with EtOH at 50 °C (ROE6) appeared most effective for preserving α-tocopherol, lutein, and squalene, yielding the highest concentrations of all three compounds. Other techniques, including mechanical press extraction and supercritical CO_2_ with EtOH as co-solvent, recovered moderate levels but did not match the antioxidant retention observed under the 50 °C solvent extraction conditions. This work focused on the composition and nutritional aspects of the examined oils. Nevertheless, in assessing an oil with respect to its potential use as a lipid source, other parameters have to be taken into account, such as the yield, the economy, and the sustainability of the process, which was beyond the aim of the present work.

*Lipidomics Results*

**Figure 2:**
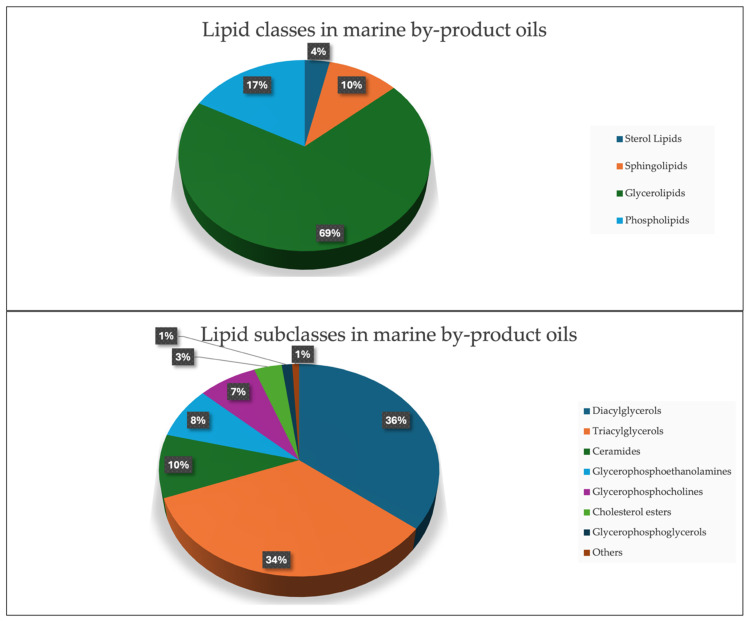
(**Up**): Pie chart showing the lipid classes presented in marine by-product oils. (**Bottom**): Pie chart showing the lipid subclasses presented in marine by-product oils.

The lipidomics analysis resulted in the detection and identification of 228 distinct lipid species across the marine oil samples, with the majority of them (69%) consisting of diglycerides (DAGs) and triglycerides (TAGs) (Figure 2), an observation consistent with multiple studies reporting TAGs and DAGs as the predominant lipid classes in marine oils. Song et al. [45] showed that TAGs constituted the dominant fraction in anchovy oils, regardless of their enrichment method, while DAGs represented the second most abundant glycerolipid class. Similarly, Windarsih et al. [46] reported that TAGs and DAGs formed the core lipid composition across five different marine fish oils, supporting their recurring prominence in marine lipidomes. Several of these lipids contained essential omega-3 and omega-6 fatty acids, which is consistent with reports showing that marine by-products frequently contain nutritionally relevant long-chain PUFAs [47,48].

Ceramides and PEs were additionally detected as relevant subclasses, in agreement with lipidomics studies that have identified these lipids in various marine tissues, including roe and shellfish [49,50,51]. A total of 102 lipid molecules were found to contain n-3 (80 compounds) and n-6 (22 compounds) fatty acids, highlighting these fish oils as a rich source of EPA and DHA, key omega-3 fatty acids known for their anti-inflammatory and cardiovascular benefits. Samples derived from squid oil exhibited a higher abundance of diglycerides and triglycerides, while roe oil samples displayed elevated levels of triglycerides containing DHA and EPA. Research supports that omega-3 and omega-6 lipids, particularly DHA and EPA, play essential roles in reducing cardiovascular risk, mitigating inflammatory responses, and promoting cognitive health [48,52,53]. These findings further substantiate the value of fish oils as a nutritionally rich supplement, especially in forms derived from roe and other marine by-products.

*Chemometric Analysis*

**Figure 3:**
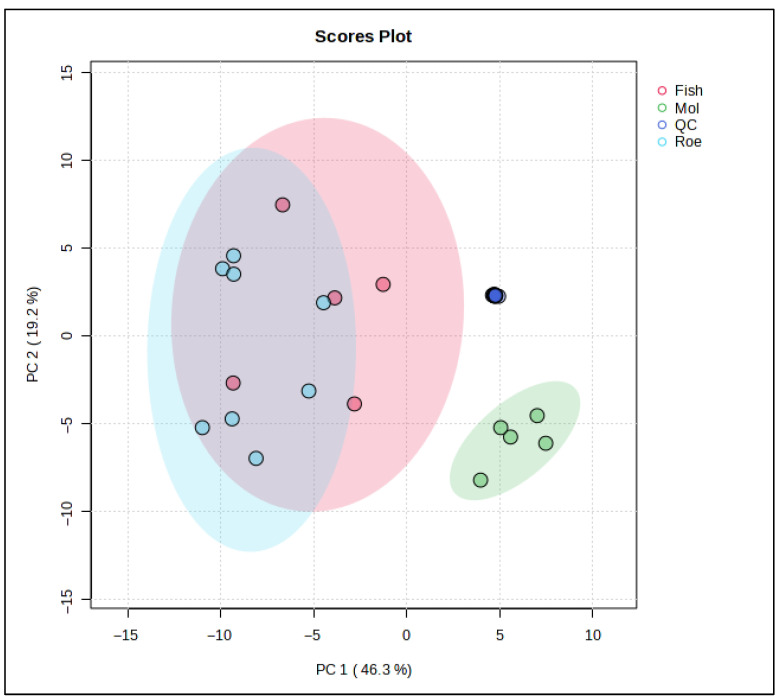
Principal Component Analysis (PCA) scores plot based on the untargeted lipidomic dataset of marine by-product oils. Samples are grouped according to biological origin: Fish (sardine heads, monkfish bellies, and anchovy heads) in red, Squid (squid bellies) in green, and Roe (grey mullet roe) in blue. PC1 and PC2 explain 46.3% and 19.2% of the total variance, respectively, highlight species driven differences in lipid composition.

To further examine the lipid differences between the marine species in this study, chemometric analyses involving both unsupervised and supervised techniques were used. Specifically, a PCA model (Figure 3) was constructed using the data obtained from the lipidomics analysis. Eight PCs explain almost 95% of the total variation between the samples, with the squid group clearly separating from the other two. Fish samples, represented by the red cluster, display a broad variance along both PC1 and PC2, suggesting heterogeneous lipidomic features within this group. The blue cluster for roe is elongated along the PC2 axis, indicating distinct lipid characteristics that diverge more in one specific compositional direction compared to fish and squid. Squid samples, represented by the green cluster, are tightly grouped, reflecting a more homogeneous lipid profile and distinct separation from both fish and roe, particularly along PC1.

These distinct clusters underscore the unique lipidomic signatures associated with each sample origin. The separation along PC1 indicates that squid lipid profiles markedly differ from fish and roe, potentially due to their unique triglyceride and fatty acid composition. Meanwhile, the positioning of squid along PC2 suggests that its lipid profile possesses specific attributes that differ from fish and roe, possibly related to structural lipids unique to its biological function.

**Figure 4:**
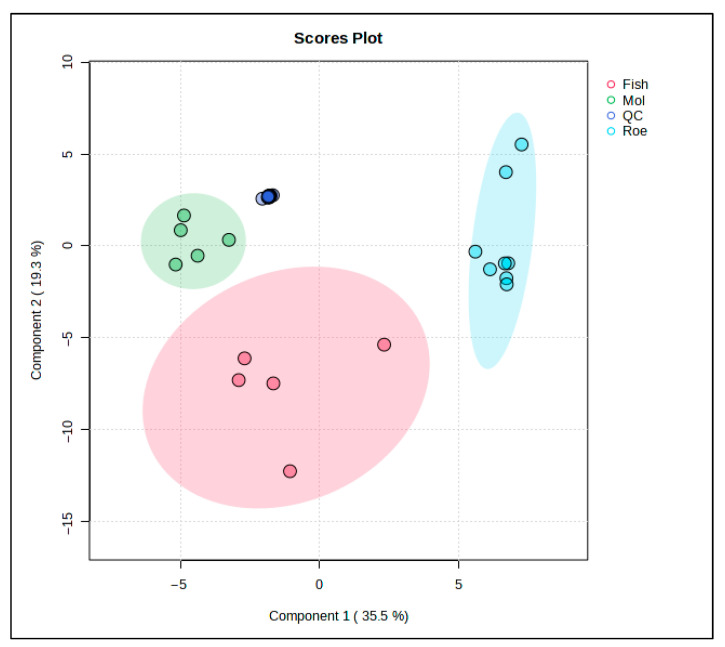
PLS-DA score plot for the comparison between fish-, squid-, and roe-derived oils.

PLS-DA was used to further study the lipid profiles of the samples. This PLS-DA scores plot (Figure 4) shows the distribution of fish, squid, roe, and QC samples, revealing distinct lipidomic patterns among these groups with the aid of supervised dimensionality reduction. Here, Component 1 explains 45.9% of the variance, while Component 3 accounts for 8.4%, collectively capturing over half of the variance in the lipid profiles within the dataset.

Fish samples, highlighted in red, occupy a broad, spread-out area along Component 1, indicating substantial lipid variability within this group. Squid samples, represented in green, form a tight cluster, suggesting a consistent lipid profile within these samples, likely reflective of their unique biochemical properties. Roe samples, in blue, are similarly distinct but form a cluster with relatively less variation along Component 3, emphasizing a unique lipid signature specific to roe tissues.

The inclusion of QC samples (blue) provides a check for the analytical consistency and reproducibility of the data. QC samples cluster closely together, indicating stability and reliability of the lipidomic measurements across the study, affirming that observed group separations are indeed due to biological differences rather than analytical variability.

This PLS-DA plot strengthens the evidence of lipidomic differentiation among fish, squid, and roe groups, with QC samples providing confidence in the robustness of the dataset. This level of group separation could potentially facilitate the identification of lipid biomarkers specific to each sample type, contributing to applications in food authentication, quality control, and dietary profiling.

**Figure 5:**
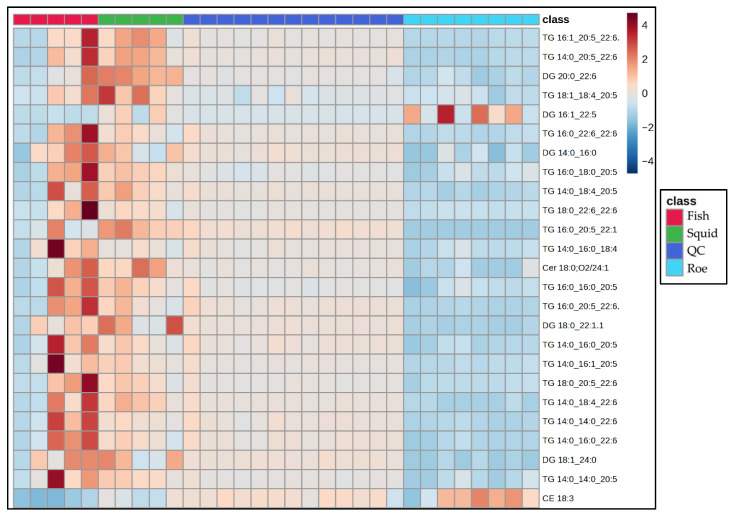
Heatmap showing the Top 25 VIPs from the PLS-DA, among the sample of marine by-product oil.

To elucidate group-specific variations in lipid profiles among fish, squid, and roe samples, we employed a heatmap visualization (Figure 5), which enables the clear representation of lipid abundance patterns across sample groups, highlighting differences in lipid composition and clustering lipid classes with similar expression trends. This heatmap provides key insights by emphasizing the relative abundance of each lipid species across groups, facilitating the identification of distinctive lipid classes that characterize each group.

The heatmap reveals several notable patterns in lipid abundance across the fish, squid, and roe sample classes. Fish samples, highlighted in red, exhibit elevated levels of specific TG and DG lipid species, as indicated by intense red coloration, suggesting a distinct lipid composition potentially tied to unique dietary or metabolic characteristics. In contrast, squid and roe samples show more moderate lipid levels, with fewer high-abundance lipids, indicating potential species-specific differences in lipid metabolism or absorption pathways. Specifically, fish oil samples exhibited a distinct lipidomic signature characterized by the significantly elevated relative abundance of multiple PUFAs containing glycerolipids, primarily within the TAG and DAG subclasses. Key differentiating species included the highly abundant TG 16:0_20:5_22:1, TG 14:0_20:5_22:6, and DG 18:1_24:0. Conversely, the roe samples demonstrated an intermediate profile, marked by an overall lower concentration of the TAG species compared to the fish group, yet were uniquely defined by the high relative intensity of DG 16:1_22:5 and the sterol ester CE 18:3. The squid oil samples profile was differentiated by uniformly low relative abundance across the majority of the major TAG species but displayed moderate enrichment in specific components like DG 20:0_22:6. These abundance patterns within the neutral lipid fraction—specifically reflecting the varying incorporation of PUFAs into TAGs and DGs—are directly linked to the species-level discrimination observed in multivariate analysis, confirming that the glycerolipid composition is a principal driver of the observed chemometric separation. The QC samples demonstrate consistent, uniform patterns, reflecting well-controlled quality assurance processes and reinforcing the reliability of the analytical results. Additionally, distinct lipid species such as CE 18:3, TG 14:0_14:0_20:5, and DG 18:1_24:0 show unique abundance profiles across the sample groups, potentially highlighting variability influenced by environmental or physiological factors. These findings underscore significant group-specific lipidomic profiles, offering insights into lipid metabolism that complement other multivariate analyses like PLS-DA.

### 3.2. Effect of Extraction Techniques on Lipid and Antioxidant Composition

Having established the species-specific lipid and antioxidant signatures in Section 3.1, we next examined how different processing approaches modify the levels of the same compounds. The extraction technique applied during marine oil recovery exerts a critical influence on both the quantitative and qualitative profiles of lipids and lipid-soluble antioxidants. In our study, extraction methods were classified into three distinct classes, solvent extraction (SE), supercritical fluid extraction combined with solvent (SFE and SE), and conventional extraction (CE), and their effects were examined through fatty acid quantification, antioxidant profiling, and multivariate lipidomic analysis.

**Figure 6:**
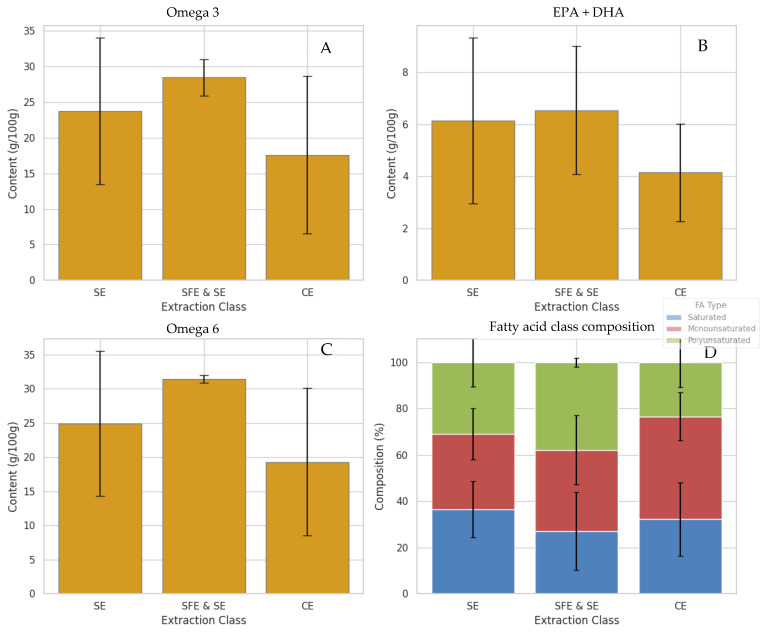
Fatty acid content in samples of marine oils based on the extraction technique used, presented as Mean ± Standard Deviation. Fatty acid composition was determined by GC-FID following transesterification of whole oil samples and is shown as extraction-class averages across the available samples. (**A**) total omega-3 content (%), (**B**) sum of EPA and DHA (%), (**C**) total omega-6 (%), and (**D**) fatty acid class composition (%).

Comparative evaluation of total EPA + DHA content (Figure 6A) revealed that both SE and SFE and SE extractions yielded similarly high levels (~27–28 g/100 g), with SFE and SE marginally outperforming SE. Conventional methods, however, exhibited significantly lower retention (~22 g/100 g), likely reflecting oxidative or hydrolytic degradation during mechanical or enzymatic processing. It should be noted, however, that these values represent averages across all available samples within each extraction class, and the dataset is not fully balanced with respect to species and extraction technique. Therefore, the observed differences reflect overall trends rather than species-independent extraction effects. A similar trend was observed for total omega-3 content (Figure 6C), with SFE and SE demonstrating the most efficient preservation, underscoring their superior capacity for retaining long-chain polyunsaturated fatty acids (PUFAs), particularly those with nutritional significance, such as EPA and DHA.

Omega-6 fatty acid levels exhibited a slightly different profile. As shown in Figure 6B, SFE and SE extractions also resulted in the highest omega-6 concentrations, followed closely by SE, whereas TE consistently yielded the lowest levels. This suggests that SFE and SE preserve not only beneficial omega-3 species but also broader classes of unsaturated lipids, including omega-6, probably due to their gentle thermodynamic conditions and improved extraction efficacy across a broader polarity range [24]. Moreover, the relative abundance of different fatty acid classes—saturated, monounsaturated, and polyunsaturated—was visualized in a compositional bar plot (Figure 6D). In this plot, it is evident that SFE and SE yielded oils with the highest proportion of PUFAs. In contrast, oils extracted through conventional methods like enzymatic hydrolysis and wet reduction showed a greater composition of saturated and MUFAs. This pattern suggests that extraction processes within the CE group may contribute to partial lipid degradation or selective retention of shorter, more saturated chains, potentially due to enzyme activity or mechanical effects such as shear stress during processing. These findings are in agreement with previous studies, which have consistently reported that SFE and SE are more effective at preserving long-chain PUFAs like EPA and DHA, while enzyme-assisted methods tend to favour MUFA retention, likely due to the lipase specificity and mild processing conditions involved [35,54,55]. Formal separation of species and extraction effects would require a fully crossed experimental design, which was beyond the scope of the present study.

In addition to lipid class composition and fatty acid profiles, the antioxidant fraction of marine oils—particularly α-tocopherol, lutein, and squalene—was significantly influenced by the extraction method applied. These compounds, while minor in concentration, play a crucial role in stabilizing polyunsaturated lipids and extending the oxidative shelf-life of marine oil products. Among the three, α-tocopherol (vitamin E) was best preserved under SFE and SE conditions, where limited oxygen exposure and controlled pressure/temperature parameters minimized degradation [24]. Oils extracted by mechanical pressing or enzymatic treatment showed the lowest tocopherol concentrations, likely due to oxidative degradation during processing. This is supported by reports of substantial tocopherol loss under oxygen-rich and heat-prone conditions common to these methods [41,42,54,56].

In contrast, moderate retention was observed in selected solvent-extracted (SE) oils, particularly when extraction was performed at lower temperatures, which minimized oxidative stress and preserved a greater proportion of tocopherols [56]. A similar pattern was observed for lutein, a carotenoid with strong antioxidant and anti-inflammatory potential. Lutein was most abundant in SFE and SE and cold SE samples, while oils obtained via conventional methods showed diminished levels, consistent with the carotenoid’s known sensitivity to heat, light, and oxidative stress. Lastly, squalene was significantly enriched in SFE and SE samples, reflecting the method’s efficiency in extracting and preserving hydrophobic bioactive compounds. SE offered variable results depending on solvent polarity and temperature, whereas CE consistently underperformed, presumably due to mechanical degradation and air exposure. Collectively, these findings reinforce that SFE and SE extractions not only optimize lipid recovery but also protect valuable minor compounds, supporting their preferential use in the production of marine oils intended for functional or nutraceutical applications.

**Figure 7:**
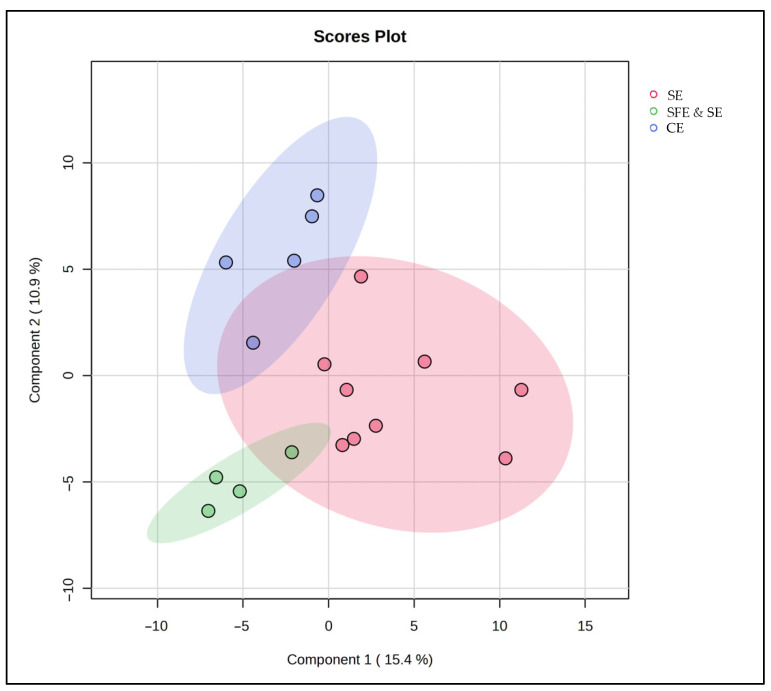
PLS-DA score plot for the comparison between SE, SFE and SE, and CE classes.

To complement the results of the FAME and antioxidant compound analyses, we next investigated whether these trends were also reflected in the full lipidomic profile using untargeted multivariate analysis. A PLS-DA model was constructed, based on the different extraction classes, and the results suggest that the extraction method has a clear impact on the lipidomic profile (Figure 7). Samples obtained via SFE and SE clustered tightly together, reflecting a high degree of biochemical consistency and extraction reproducibility. CE-extracted samples also formed a distinct, cohesive group, highlighting a different lipidomic composition from the other extraction techniques. In contrast, SE-extracted samples were more scattered, highlighting considerable variability within this group. This dispersion is likely due to the broad nature of the SE category, which included extractions at both 25 °C and 50 °C—conditions known to influence lipid yield and stability. Moreover, the partial overlaps between extraction groups suggest that lipid composition is not solely determined by the extraction technique but also influenced by the marine species used. Shared species-specific lipid traits likely contributed to similarities between certain samples, regardless of the extraction technique used for their production.

**Figure 8:**
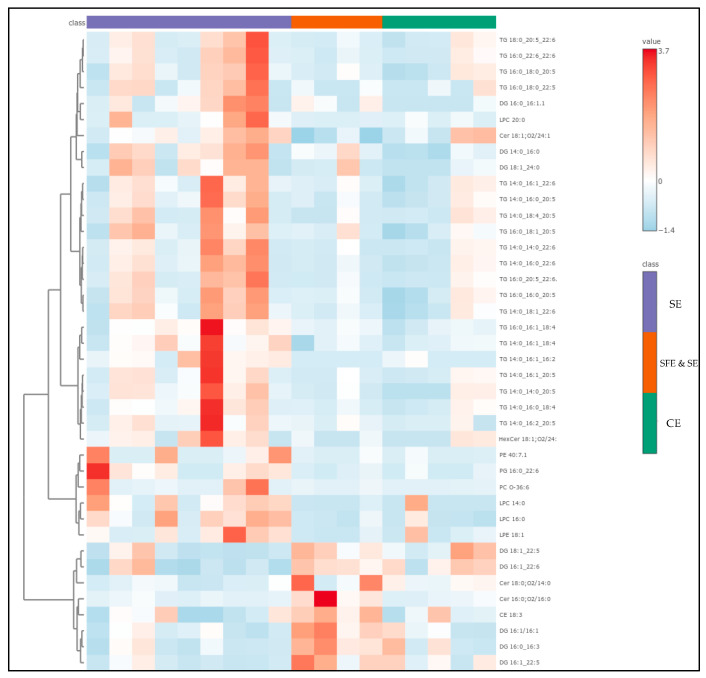
Heatmap showing the top 40 VIPs from the PLS-DA, among the samples of different extraction techniques.

The compounds with the highest VIP scores—representing the most influential lipids in distinguishing between extraction classes—are listed in Table S3 of the ESM. From this group, the top 40 were used to generate the heatmap shown in Figure 8, highlighting the lipid species most responsible for the observed sample clustering. Interestingly, half of these key differentiators were TGs—complex storage lipids that often carry high-value omega-3 fatty acids such as EPA and DHA. These were most abundant in the SFE and SE extracts, reinforcing the idea that this technique is more effective at preserving intact, functional lipids. DGs accounted for another 20% of the VIPs and were more prevalent in TE and SE samples, where they likely resulted from partial hydrolysis or TG degradation [54,55,57]. Similarly, lysophosphatidylcholines (LPCs)—well-known markers of membrane damage and oxidative stress—were also more common in these conventional and solvent-based extracts. Only a few phosphatidylethanolamines (PEs) appeared among the VIPs, but those detected were DHA-rich and notably concentrated in SFE and SE samples.

The heatmap visualization of these top VIP lipids added further clarity to the abovementioned results. Samples clustered according to extraction method, reinforcing the trends seen in the PLS-DA analysis. SFE and SE samples stood out, marked by high levels of DHA- and EPA-containing triglycerides and phospholipids. In contrast, CE-extracted samples were dominated by LPCs and DGs, indicative of greater oxidative and mechanical stress during processing. The SE group again proved to be more complex: some SE samples aligned closely with SFE profiles—likely those extracted at optimized conditions—while others mirrored the degradation-prone TE group. Together, these findings paint a detailed picture of how different extraction methods influence not just the quantity, but also the quality and complexity of lipids in marine oils. SFE and SE emerge as the superior technique, offering the best preservation of long-chain, bioactive omega-3 lipids and the lowest abundance of degradation markers. In contrast, CE and conventional SE methods appear to compromise lipid integrity to varying degrees. These lipidomic insights reinforce the importance of choosing the right extraction strategy, especially when targeting high-value nutritional and functional compounds. For producers aiming to deliver potent, stable, and bioactive marine lipid formulations, SFE and SE represent the most promising and scientifically supported approach.

## 4. Conclusions

This study comprehensively assessed how different extraction techniques influence the lipid composition and antioxidant content of oils derived from various marine by-products. The results clearly demonstrate that both the extraction method and the biological origin of the raw material significantly impact the nutritional and functional properties of the final oil product, particularly through differences in PUFA-rich triglycerides (TAGs), diglycerides (DGs), and key antioxidant molecules. Among the extraction methods examined, the supercritical extraction with CO_2_ and ethanol as co-solvents (SFE-SE) emerged as the most effective in preserving bioactive and antioxidant lipid compounds. Oils obtained through this method showed consistently higher levels of essential omega-3 fatty acids—particularly EPA and DHA—as well as elevated concentrations of antioxidant molecules, such as α-tocopherol, lutein, and squalene. These compounds are critical for cardiovascular, neurological, and immune health, and their presence substantially enhances the nutritional value and oxidative stability of marine oils. In contrast, conventional extraction methods, such as wet reduction and mechanical pressing, resulted in lower retention of polyunsaturated fatty acids and antioxidants and a higher presence of hydrolysis/oxidation-associated lipids (e.g., DGs and LPCs), likely due to increased exposure to heat, oxygen, or enzymatic degradation during processing. Solvent-based extractions offered variable results, with temperature playing a decisive role in lipid and antioxidant preservation. Notably, roe-derived oils consistently demonstrated superior lipid profiles across all analyses, including higher levels of long-chain polyunsaturated fats and antioxidant compounds. This finding highlights grey mullet roe as a particularly rich and under-utilized source of health-promoting marine lipids, reinforcing its potential for use in high-value nutraceutical and cosmeceutical formulations. The application of untargeted lipidomics, coupled with advanced chemometric models (PCA and PLS-DA), further confirmed the clear biochemical separation between sample groups based on both species and extraction class. Discriminating variables included PUFA-rich TAGs and DAGs in fish oils and characteristic DG and sterol ester species in grey mullet roe and squid belly samples. This analytical approach not only provided a detailed molecular fingerprint of each sample but also revealed biomarkers that could guide future quality control, traceability, and product development efforts.

Overall, the findings presented in this work highlight how strongly the choice of extraction method shapes the nutritional quality and functional value of marine-derived oils. Optimizing conditions—particularly through supercritical CO_2_–ethanol (SFE-SE)—can significantly enhance both the yield and preservation of key bioactive lipids. This work also strengthens the case for marine by-products as a viable, sustainable source of high-value compounds, supporting their integration into circular bioeconomy models and value-added applications.

## Figures and Tables

**Table 2:** Lutein, α-tocopherol, and squalene results of the analyzed samples *. Sample codes indicate species and tissue origin.

Sample	Lutein (mg/Kg)	a-Tocopherol (mg/Kg)	Squalene (mg/Kg)
ROE1	50	345	2075
ROE2	43	205	2604
ROE3	88	354	n.d.
ROE4	103	298	n.d.
ROE5	10	440	1301
ROE6	125	469	6049
ROE7	124	307	n.d.
SAR1	87	363	n.d.
SAR2	75	312	n.d.
ANC1	n.d.	n.d.	1004
ANC2	n.d.	n.d.	4120
MON1	n.d.	n.d.	2419
SQD1	n.d.	n.d.	n.d.
SQD2	n.d.	n.d.	n.d.
SQD3	n.d.	n.d.	n.d.
SQD4	n.d.	n.d.	n.d.

* only samples where the abovementioned compounds were detected are presented. Sample codes indicate species group and extraction replicate: ROE = grey mullet roe (Mugil cephalus), SAR = sardine heads (Sardina pilchardus), ANC = anchovy heads (Engraulis encrasicolus), MON = monkfish bellies (Lophius piscatorius), and SQD = squid bellies (Nototodarus sloanii). n.d. = non-detected.

## Data Availability

The original contributions presented in this study are included in the article/supplementary material. Further inquiries can be directed to the corresponding author.

## Supplementary Materials

The following supporting information can be downloaded at https://www.mdpi.com/article/10.3390/antiox15010095/s1: Table S1. List of samples and sample information; Table S2. Lipid compounds detected in marine oils with their molecular formula, lipid class, and subclass; Table S3. List of VIP compounds with VIP score > 1 found in marine oils.
