# Supplementary material for: Lipidomic Characterization of Marine By-Product Oils: Impact of Species and Extraction Methods on Lipid Profile and Antioxidant Potential
Source: Antioxidants (Basel). 2026 Jan 12;15(1):95. doi: 10.3390/antiox15010095 (PMC12838132; doi:10.3390/antiox15010095)
Supplement: Supplementary file 1 [file antioxidants-15-00095-s001.zip › antioxidants-4006665-supplementary.pdf]

Electronic Supplementary Material

Lipidomic Characterization of Marine By-Product Oils: Impact of Species and Extraction Methods on Lipid Profile and Antioxidant Potential

Ioannis C. Martakos[1], Paraskeui Tzika[2], Marilena E. Dasenaki[3], Eleni P. Kalogianni[2] and Nikolaos S. Thomaidis[1,*]

[1]Laboratory of Analytical Chemistry, Dept. of Chemistry, National and Kapodistrian University of Athens, Panepistimiopolis Zographou; johnmrtk@chem.uoa.gr; ntho@chem.uoa.gr

[2] Department of Food Science and Technology, International Hellenic University, 57400, Thessaloniki, Greece; elekalo@ihu.gr; tzikap@food.ihu.gr

[3] Laboratory of Food Chemistry, Dept. of Chemistry, National and Kapodistrian University of Athens, Panepistimiopolis Zographou; mdasenaki@chem.uoa.gr

* Correspondence:
Nikolaos S. Thomaidis; E-mail: ntho@chem.uoa.gr; Tel.: +30 2107274317

Electronic Supplementary Material

Table of contents

Table S1. List of samples and sample information S3

Table S2. Lipid compounds detected in marine oils with their molecular formula, lipid class and subclass S4

Table S3. List of VIP compounds with VIP score > 1 found in marine oils S9

Table S1. List of samples and sample information

Sample Name	Species	Extraction Technique	Parameters	Class
ANC1	Fish	Solvent Extraction	ethanol 25 oC	SE
ANC2	Fish	Solvent Extraction	ethanol 50 oC	SE
MON1	Fish	Solvent Extraction	ethanol 50 oC	SE
SAR1	Fish	Solvent Extraction	ethanol 25 oC	SE
SAR2	Fish	Solvent Extraction	ethanol 50 oC	SE
ROE1	Roe	Super Critical Fluid Extraction	CO_2 & ethanol 37 oC	SFE & SE
ROE2	Roe	Super Critical Fluid Extraction	CO_2 & ethanol 50 oC	SFE & SE
ROE3	Roe	Mechanical Press	-	TE
ROE4	Roe	Wet Reduction	-	TE
ROE5	Roe	Solvent Extraction	hexane/ethanol 25 oC	SE
ROE6	Roe	Solvent Extraction	ethanol 25 oC	SE
ROE7	Roe	Mechanical Press & Solvent Extraction	ethanol 50 oC	TE
SQD1	Squid	Super Critical Fluid Extraction	CO_2 & ethanol 40 oC	SFE & SE
SQD2	Squid	Enzymatically assisted extraction	Alcalase ® 1% 2h	TE
SQD3	Squid	Enzymatically assisted extraction	Protease ® 0.5% 1h	TE
SQD4	Squid	Solvent Extraction	ethanol 50 oC	SE

Table S2. Lipid compounds detected in marine oils with their molecular formula, lipid class and subclass

Name	Molecular Formula	Lipid Class	Lipid Subclass
**CE 14:0**	C41H72O2	Sterol Lipids	Cholesterol esters
**CE 16:2**	C43H72O2	Sterol Lipids	Cholesterol esters
**CE 16:3**	C43H70O2	Sterol Lipids	Cholesterol esters
**CE 16:4**	C43H68O2	Sterol Lipids	Cholesterol esters
**CE 18:2**	C45H76O2	Sterol Lipids	Cholesterol esters
**CE 18:3**	C45H74O2	Sterol Lipids	Cholesterol esters
**CE 18:4**	C45H72O2	Sterol Lipids	Cholesterol esters
**CE 20:4**	C47H76O2	Sterol Lipids	Cholesterol esters
**Cer 14:1;O2/18:1**	C32H61NO3	Sphingolipids	Ceramides
**Cer 14:1;O2/22:6**	C36H59NO3	Sphingolipids	Ceramides
**Cer 16:0;O2/16:0**	C32H65NO3	Sphingolipids	Ceramides
**Cer 16:0;O2/22:1**	C38H75NO3	Sphingolipids	Ceramides
**Cer 16:1;O2/16:0**	C32H63NO3	Sphingolipids	Ceramides
**Cer 16:1;O2/20:0**	C36H71NO3	Sphingolipids	Ceramides
**Cer 16:1;O2/22:1**	C38H73NO3	Sphingolipids	Ceramides
**Cer 16:1;O2/22:2**	C38H71NO3	Sphingolipids	Ceramides
**Cer 16:1;O2/24:1**	C40H77NO3	Sphingolipids	Ceramides
**Cer 16:1;O2/26:4**	C42H75NO3	Sphingolipids	Ceramides
**Cer 17:0;O2/15:0;O**	C32H65NO4	Sphingolipids	Ceramides
**Cer 18:0;O2/14:0**	C32H65NO3	Sphingolipids	Ceramides
**Cer 18:0;O2/24:1**	C42H83NO3	Sphingolipids	Ceramides
**Cer 18:1;O2/16:0**	C34H67NO3	Sphingolipids	Ceramides
**Cer 18:1;O2/22:0;O**	C40H79NO4	Sphingolipids	Ceramides
**Cer 18:1;O2/24:0;O**	C42H83NO4	Sphingolipids	Ceramides
**Cer 18:1;O2/24:1**	C42H81NO3	Sphingolipids	Ceramides
**Cer 18:2;O2/16:0**	C34H65NO3	Sphingolipids	Ceramides
**Cer 18:3;O2/14:0**	C32H59NO3	Sphingolipids	Ceramides
**Cer 18:3;O2/24:1**	C42H77NO3	Sphingolipids	Ceramides
**Cer 18:3;O2/24:1;O**	C42H77NO4	Sphingolipids	Ceramides
**Cer 20:2;O2/16:0**	C36H69NO3	Sphingolipids	Ceramides
**DG 12:0_16:1**	C31H58O5	Glycerolipids	Diacylglycerols
**DG 14:0_16:0**	C33H64O5	Glycerolipids	Diacylglycerols
**DG 14:0_16:1**	C33H62O5	Glycerolipids	Diacylglycerols
**DG 14:0_18:3**	C35H62O5	Glycerolipids	Diacylglycerols
**DG 14:0_18:4**	C35H60O5	Glycerolipids	Diacylglycerols
**DG 14:0_20:4**	C37H64O5	Glycerolipids	Diacylglycerols
**DG 14:0_20:5**	C37H62O5	Glycerolipids	Diacylglycerols
**DG 14:0_22:6**	C39H64O5	Glycerolipids	Diacylglycerols
**DG 16:0/16:0**	C35H68O5	Glycerolipids	Diacylglycerols
**DG 16:0_16:1**	C35H66O5	Glycerolipids	Diacylglycerols
**DG 16:0_16:1**	C35H66O5	Glycerolipids	Diacylglycerols
**DG 16:0_16:2**	C35H64O5	Glycerolipids	Diacylglycerols
**DG 16:0_16:3**	C35H62O5	Glycerolipids	Diacylglycerols
**DG 16:0_16:4**	C35H60O5	Glycerolipids	Diacylglycerols

**DG 16:0_18:0**	C37H72O5	Glycerolipids	Diacylglycerols
**DG 16:0_18:1**	C37H70O5	Glycerolipids	Diacylglycerols
**DG 16:0_18:3**	C37H66O5	Glycerolipids	Diacylglycerols
**DG 16:0_18:4**	C37H64O5	Glycerolipids	Diacylglycerols
**DG 16:0_20:1**	C39H74O5	Glycerolipids	Diacylglycerols
**DG 16:0_20:4**	C39H68O5	Glycerolipids	Diacylglycerols
**DG 16:0_20:4**	C39H68O5	Glycerolipids	Diacylglycerols
**DG 16:0_20:5**	C39H66O5	Glycerolipids	Diacylglycerols
**DG 16:0_22:1**	C41H78O5	Glycerolipids	Diacylglycerols
**DG 16:0_22:6**	C41H68O5	Glycerolipids	Diacylglycerols
**DG 16:1/16:1**	C35H64O5	Glycerolipids	Diacylglycerols
**DG 16:1_16:4**	C35H58O5	Glycerolipids	Diacylglycerols
**DG 16:1_18:1**	C37H68O5	Glycerolipids	Diacylglycerols
**DG 16:1_18:3**	C37H64O5	Glycerolipids	Diacylglycerols
**DG 16:1_18:4**	C37H62O5	Glycerolipids	Diacylglycerols
**DG 16:1_20:4**	C39H66O5	Glycerolipids	Diacylglycerols
**DG 16:1_20:5**	C39H64O5	Glycerolipids	Diacylglycerols
**DG 16:1_22:5**	C41H68O5	Glycerolipids	Diacylglycerols
**DG 16:1_22:6**	C41H66O5	Glycerolipids	Diacylglycerols
**DG 16:2_20:5**	C39H62O5	Glycerolipids	Diacylglycerols
**DG 16:2_22:6**	C41H64O5	Glycerolipids	Diacylglycerols
**DG 18:0/18:0**	C39H76O5	Glycerolipids	Diacylglycerols
**DG 18:0_20:1**	C41H78O5	Glycerolipids	Diacylglycerols
**DG 18:0_20:4**	C41H72O5	Glycerolipids	Diacylglycerols
**DG 18:0_20:5**	C41H70O5	Glycerolipids	Diacylglycerols
**DG 18:0_22:1**	C43H82O5	Glycerolipids	Diacylglycerols
**DG 18:0_22:1**	C43H82O5	Glycerolipids	Diacylglycerols
**DG 18:0_22:6**	C43H72O5	Glycerolipids	Diacylglycerols
**DG 18:1_18:2**	C39H70O5	Glycerolipids	Diacylglycerols
**DG 18:1_18:3**	C39H68O5	Glycerolipids	Diacylglycerols
**DG 18:1_18:4**	C39H66O5	Glycerolipids	Diacylglycerols
**DG 18:1_20:0**	C41H78O5	Glycerolipids	Diacylglycerols
**DG 18:1_20:1**	C41H76O5	Glycerolipids	Diacylglycerols
**DG 18:1_20:3**	C41H72O5	Glycerolipids	Diacylglycerols
**DG 18:1_20:4**	C41H70O5	Glycerolipids	Diacylglycerols
**DG 18:1_20:5**	C41H68O5	Glycerolipids	Diacylglycerols
**DG 18:1_22:5**	C43H72O5	Glycerolipids	Diacylglycerols
**DG 18:1_22:6**	C43H70O5	Glycerolipids	Diacylglycerols
**DG 18:1_24:0**	C45H86O5	Glycerolipids	Diacylglycerols
**DG 18:2_20:1**	C41H74O5	Glycerolipids	Diacylglycerols
**DG 18:2_20:5**	C41H66O5	Glycerolipids	Diacylglycerols
**DG 18:2_22:1**	C43H78O5	Glycerolipids	Diacylglycerols
**DG 18:2_22:6**	C43H68O5	Glycerolipids	Diacylglycerols
**DG 18:3_20:5**	C41H64O5	Glycerolipids	Diacylglycerols
**DG 18:3_22:5**	C43H68O5	Glycerolipids	Diacylglycerols
**DG 20:0_20:5**	C43H74O5	Glycerolipids	Diacylglycerols
**DG 20:0_22:6**	C45H76O5	Glycerolipids	Diacylglycerols

**DG 20:1/20:1**	C43H80O5	Glycerolipids	Diacylglycerols
**DG 20:1_20:4**	C43H74O5	Glycerolipids	Diacylglycerols
**DG 20:1_20:5**	C43H72O5	Glycerolipids	Diacylglycerols
**DG 20:1_22:1**	C45H84O5	Glycerolipids	Diacylglycerols
**DG 20:1_22:5**	C45H76O5	Glycerolipids	Diacylglycerols
**DG 20:1_22:6**	C45H74O5	Glycerolipids	Diacylglycerols
**DG 20:1_24:1**	C47H88O5	Glycerolipids	Diacylglycerols
**DG 20:3_22:1**	C45H80O5	Glycerolipids	Diacylglycerols
**DG 20:4_22:1**	C45H78O5	Glycerolipids	Diacylglycerols
**DG 20:5_22:1**	C45H76O5	Glycerolipids	Diacylglycerols
**DG 20:5_24:1**	C47H80O5	Glycerolipids	Diacylglycerols
**DG 22:1_22:5**	C47H80O5	Glycerolipids	Diacylglycerols
**DG 22:1_22:6**	C47H78O5	Glycerolipids	Diacylglycerols
**DG O-28:0**	C31H62O4	Glycerolipids	Diacylglycerols
**DG O-28:1**	C31H60O4	Glycerolipids	Diacylglycerols
**DG O-32:0**	C35H70O4	Glycerolipids	Diacylglycerols
**DG O-34:1**	C37H72O4	Glycerolipids	Diacylglycerols
**DG O-36:3**	C39H72O4	Glycerolipids	Diacylglycerols
**DG O-36:6**	C39H66O4	Glycerolipids	Diacylglycerols
**DG O-40:8**	C43H70O4	Glycerolipids	Diacylglycerols
**HexCer 18:1;O2/24:1**	C48H91NO8	Sphingolipids	Neutral Glycosphingolipids
**LPC 14:0**	C22H46NO7P	Phospholipids	Glycerophosphocholines
**LPC 16:0**	C24H50NO7P	Phospholipids	Glycerophosphocholines
**LPC 18:0**	C26H54NO7P	Phospholipids	Glycerophosphocholines
**LPC 18:1**	C26H52NO7P	Phospholipids	Glycerophosphocholines
**LPC 18:2**	C26H50NO7P	Phospholipids	Glycerophosphocholines
**LPC 20:0**	C28H58NO7P	Phospholipids	Glycerophosphocholines
**LPE 18:1**	C23H46NO7P	Phospholipids	Glycerophosphoethanolamines
**LPE 20:1**	C25H50NO7P	Phospholipids	Glycerophosphoethanolamines
**PC 16:0_20:5**	C44H78NO8P	Phospholipids	Glycerophosphocholines
**PC 16:0_22:6**	C46H80NO8P	Phospholipids	Glycerophosphocholines
**PC 32:2**	C40H76NO8P	Phospholipids	Glycerophosphocholines
**PC 34:2**	C42H80NO8P	Phospholipids	Glycerophosphocholines
**PC 34:4**	C42H76NO8P	Phospholipids	Glycerophosphocholines
**PC 34:5**	C42H74NO8P	Phospholipids	Glycerophosphocholines
**PC 36:5**	C44H78NO8P	Phospholipids	Glycerophosphocholines
**PC 40:8**	C48H80NO8P	Phospholipids	Glycerophosphocholines
**PC O-36:6**	C44H78NO7P	Phospholipids	Glycerophosphocholines
**PC O-38:7**	C46H80NO7P	Phospholipids	Glycerophosphocholines
**PC O-42:7**	C50H88NO7P	Phospholipids	Glycerophosphocholines
**PE 16:0_22:6**	C43H74NO8P	Phospholipids	Glycerophosphoethanolamines
**PE 36:5**	C41H72NO8P	Phospholipids	Glycerophosphoethanolamines
**PE 36:6**	C41H70NO8P	Phospholipids	Glycerophosphoethanolamines
**PE 38:7**	C43H72NO8P	Phospholipids	Glycerophosphoethanolamines
**PE 40:6**	C45H78NO8P	Phospholipids	Glycerophosphoethanolamines
**PE 40:7**	C45H76NO8P	Phospholipids	Glycerophosphoethanolamines
**PE 40:7**	C45H76NO8P	Phospholipids	Glycerophosphoethanolamines

**PE 40:7**	C45H76NO8P	Phospholipids	Glycerophosphoethanolamines
**PE 40:8**	C45H74NO8P	Phospholipids	Glycerophosphoethanolamines
**PE 40:8**	C45H74NO8P	Phospholipids	Glycerophosphoethanolamines
**PE 40:8**	C45H74NO8P	Phospholipids	Glycerophosphoethanolamines
**PE 42:8**	C47H78NO8P	Phospholipids	Glycerophosphoethanolamines
**PE 42:8**	C47H78NO8P	Phospholipids	Glycerophosphoethanolamines
**PE O-36:5**	C41H74NO7P	Phospholipids	Glycerophosphoethanolamines
**PE O-38:5**	C43H78NO7P	Phospholipids	Glycerophosphoethanolamines
**PE O-38:6**	C43H76NO7P	Phospholipids	Glycerophosphoethanolamines
**PG 16:0_20:5**	C42H73O10P	Phospholipids	Glycerophosphoglycerols
**PG 16:0_22:6**	C44H75O10P	Phospholipids	Glycerophosphoglycerols
**PG 16:1_22:6**	C44H73O10P	Phospholipids	Glycerophosphoglycerols
**PI 38:4**	C47H83O13P	Phospholipids	Glycerophosphoinositols
**TG 14:0_14:0_20:5**	C51H88O6	Glycerolipids	Triacylglycerols
**TG 14:0_14:0_22:6**	C53H90O6	Glycerolipids	Triacylglycerols
**TG 14:0_16:0_16:1**	C49H92O6	Glycerolipids	Triacylglycerols
**TG 14:0_16:0_16:2**	C49H90O6	Glycerolipids	Triacylglycerols
**TG 14:0_16:0_18:4**	C51H90O6	Glycerolipids	Triacylglycerols
**TG 14:0_16:0_20:5**	C53H92O6	Glycerolipids	Triacylglycerols
**TG 14:0_16:0_22:1**	C55H104O6	Glycerolipids	Triacylglycerols
**TG 14:0_16:0_22:6**	C55H94O6	Glycerolipids	Triacylglycerols
**TG 14:0_16:1_16:1**	C49H90O6	Glycerolipids	Triacylglycerols
**TG 14:0_16:1_16:2**	C49H88O6	Glycerolipids	Triacylglycerols
**TG 14:0_16:1_18:4**	C51H88O6	Glycerolipids	Triacylglycerols
**TG 14:0_16:1_20:5**	C53H90O6	Glycerolipids	Triacylglycerols
**TG 14:0_16:1_22:6**	C55H92O6	Glycerolipids	Triacylglycerols
**TG 14:0_16:2_20:5**	C53H88O6	Glycerolipids	Triacylglycerols
**TG 14:0_16:4_20:5**	C53H84O6	Glycerolipids	Triacylglycerols
**TG 14:0_18:1_22:6**	C57H96O6	Glycerolipids	Triacylglycerols
**TG 14:0_18:4_20:5**	C55H88O6	Glycerolipids	Triacylglycerols
**TG 14:0_18:4_22:6**	C57H90O6	Glycerolipids	Triacylglycerols
**TG 14:0_20:5_22:6**	C59H92O6	Glycerolipids	Triacylglycerols
**TG 16:0_16:0_16:1**	C51H96O6	Glycerolipids	Triacylglycerols
**TG 16:0_16:0_18:1**	C53H100O6	Glycerolipids	Triacylglycerols
**TG 16:0_16:0_18:4**	C53H94O6	Glycerolipids	Triacylglycerols
**TG 16:0_16:0_20:4**	C55H98O6	Glycerolipids	Triacylglycerols
**TG 16:0_16:0_20:5**	C55H96O6	Glycerolipids	Triacylglycerols
**TG 16:0_16:0_22:6**	C57H98O6	Glycerolipids	Triacylglycerols
**TG 16:0_16:1_16:1**	C51H94O6	Glycerolipids	Triacylglycerols
**TG 16:0_16:1_18:1**	C53H98O6	Glycerolipids	Triacylglycerols
**TG 16:0_16:1_18:2**	C53H96O6	Glycerolipids	Triacylglycerols
**TG 16:0_16:1_18:4**	C53H92O6	Glycerolipids	Triacylglycerols
**TG 16:0_16:1_22:5**	C57H98O6	Glycerolipids	Triacylglycerols
**TG 16:0_18:0_20:1**	C57H108O6	Glycerolipids	Triacylglycerols
**TG 16:0_18:0_20:4**	C57H102O6	Glycerolipids	Triacylglycerols
**TG 16:0_18:0_20:5**	C57H100O6	Glycerolipids	Triacylglycerols
**TG 16:0_18:0_22:5**	C59H104O6	Glycerolipids	Triacylglycerols

**TG 16:0_18:1_18:1**	C55H102O6	Glycerolipids	Triacylglycerols
**TG 16:0_18:1_18:4**	C55H96O6	Glycerolipids	Triacylglycerols
**TG 16:0_18:1_20:1**	C57H106O6	Glycerolipids	Triacylglycerols
**TG 16:0_18:1_20:5**	C57H98O6	Glycerolipids	Triacylglycerols
**TG 16:0_18:1_22:5**	C59H102O6	Glycerolipids	Triacylglycerols
**TG 16:0_18:1_22:6**	C59H100O6	Glycerolipids	Triacylglycerols
**TG 16:0_18:4_20:1**	C57H100O6	Glycerolipids	Triacylglycerols
**TG 16:0_18:4_22:1**	C59H104O6	Glycerolipids	Triacylglycerols
**TG 16:0_20:1_20:1**	C59H110O6	Glycerolipids	Triacylglycerols
**TG 16:0_20:1_20:5**	C59H102O6	Glycerolipids	Triacylglycerols
**TG 16:0_20:1_22:5**	C61H106O6	Glycerolipids	Triacylglycerols
**TG 16:0_20:1_22:6**	C61H104O6	Glycerolipids	Triacylglycerols
**TG 16:0_20:4_22:1**	C61H108O6	Glycerolipids	Triacylglycerols
**TG 16:0_20:5_22:1**	C61H106O6	Glycerolipids	Triacylglycerols
**TG 16:0_20:5_22:6**	C61H96O6	Glycerolipids	Triacylglycerols
**TG 16:0_20:5_22:6**	C61H96O6	Glycerolipids	Triacylglycerols
**TG 16:0_22:1_22:6**	C63H108O6	Glycerolipids	Triacylglycerols
**TG 16:0_22:6_22:6**	C63H98O6	Glycerolipids	Triacylglycerols
**TG 16:1_16:1_18:1**	C53H96O6	Glycerolipids	Triacylglycerols
**TG 16:1_16:2_18:1**	C53H94O6	Glycerolipids	Triacylglycerols
**TG 16:1_16:4_20:5**	C55H86O6	Glycerolipids	Triacylglycerols
**TG 16:1_18:1_18:1**	C55H100O6	Glycerolipids	Triacylglycerols
**TG 16:1_18:1_20:1**	C57H104O6	Glycerolipids	Triacylglycerols
**TG 16:1_18:1_20:5**	C57H96O6	Glycerolipids	Triacylglycerols
**TG 16:1_18:1_22:6**	C59H98O6	Glycerolipids	Triacylglycerols
**TG 16:1_18:4_20:5**	C57H90O6	Glycerolipids	Triacylglycerols
**TG 16:1_20:5_22:6**	C61H94O6	Glycerolipids	Triacylglycerols
**TG 16:1_20:5_22:6**	C61H94O6	Glycerolipids	Triacylglycerols
**TG 16:1_22:5_22:6**	C63H98O6	Glycerolipids	Triacylglycerols
**TG 18:0_20:5_22:6**	C63H100O6	Glycerolipids	Triacylglycerols
**TG 18:0_22:6_22:6**	C65H102O6	Glycerolipids	Triacylglycerols
**TG 18:1_18:1_20:1**	C59H108O6	Glycerolipids	Triacylglycerols
**TG 18:1_18:1_20:5**	C59H100O6	Glycerolipids	Triacylglycerols
**TG 18:1_18:4_20:5**	C59H94O6	Glycerolipids	Triacylglycerols
**TG 18:1_20:1_20:1**	C61H112O6	Glycerolipids	Triacylglycerols
**TG 18:1_20:1_20:5**	C61H104O6	Glycerolipids	Triacylglycerols
**TG 18:1_20:1_22:6**	C63H106O6	Glycerolipids	Triacylglycerols
**TG 18:1_20:5_22:6**	C63H98O6	Glycerolipids	Triacylglycerols
**TG 18:1_22:5_22:6**	C65H102O6	Glycerolipids	Triacylglycerols
**TG 18:2_20:1_22:6**	C63H104O6	Glycerolipids	Triacylglycerols
**TG 20:1_20:1_20:5**	C63H108O6	Glycerolipids	Triacylglycerols
**TG 20:1_20:5_22:5**	C65H104O6	Glycerolipids	Triacylglycerols
**TG 20:1_20:5_22:6**	C65H102O6	Glycerolipids	Triacylglycerols

Table S3. List of VIP compounds with VIP score > 1 found in marine oils

	Comp. 1	Comp. 2	Comp. 3	Comp. 4	Comp. 5	Comp. 6	Comp. 7	Comp. 8
**LPC 16:0**	2.44	2.18	2.07	2.0	1.86	1.79	1.78	1.78
**DG 16:1_22:5**	2.27	2.07	1.96	1.9	1.76	1.71	1.7	1.7
**DG 16:1_22:6**	2.07	1.84	1.81	1.76	1.63	1.56	1.56	1.56
**DG 16:0_16:3**	2.07	1.91	1.83	1.77	1.65	1.58	1.57	1.57
**DG 18:1_22:5**	2.02	1.79	1.73	1.68	1.57	1.53	1.52	1.52
**TG 14:0_16:1_16:2**	2.01	1.78	1.66	1.6	1.57	1.5	1.49	1.5
**TG 14:0_18:1_22:6**	1.97	1.75	1.68	1.63	1.52	1.46	1.45	1.45
**DG 16:1/16:1**	1.96	1.91	1.82	1.76	1.63	1.57	1.56	1.56
**LPC 14:0**	1.94	1.72	1.68	1.62	1.54	1.53	1.52	1.51
**PG 16:0_22:6**	1.94	1.74	1.83	1.78	1.79	1.71	1.7	1.7
**Cer 18:0;O2/14:0**	1.93	1.76	1.68	1.63	1.55	1.49	1.48	1.48
**TG 14:0_16:1_18:4**	1.92	1.7	1.59	1.54	1.53	1.56	1.55	1.55
**TG 14:0_14:0_20:5**	1.86	1.66	1.6	1.55	1.44	1.38	1.38	1.38
**TG 14:0_16:0_22:6**	1.85	1.64	1.6	1.55	1.44	1.4	1.39	1.39
**TG 16:0_20:5_22:6.1**	1.8	1.59	1.56	1.51	1.41	1.36	1.36	1.35
**TG 14:0_16:0_20:5**	1.76	1.56	1.55	1.5	1.4	1.34	1.33	1.33
**TG 16:0_16:0_20:5**	1.75	1.56	1.55	1.49	1.39	1.33	1.32	1.32
**TG 14:0_14:0_22:6**	1.69	1.49	1.52	1.48	1.38	1.33	1.32	1.32
**LPE 18:1**	1.68	1.5	1.4	1.36	1.27	1.22	1.22	1.21
**LPC 20:0**	1.68	1.49	1.4	1.35	1.26	1.28	1.29	1.28
**CE 18:3**	1.67	1.62	1.53	1.49	1.38	1.4	1.39	1.4
**PE 40:7.1**	1.66	1.5	1.67	1.62	1.53	1.47	1.47	1.47
**DG 18:1_24:0**	1.66	1.53	1.43	1.4	1.34	1.38	1.37	1.37
**TG 16:0_18:1_20:5**	1.63	1.49	1.45	1.4	1.31	1.28	1.27	1.27
**TG 16:0_18:0_22:5**	1.63	1.45	1.4	1.35	1.27	1.32	1.34	1.34
**TG 16:0_22:6_22:6**	1.61	1.43	1.43	1.38	1.3	1.29	1.28	1.28
**Cer 16:0;O2/16:0**	1.59	1.62	1.54	1.49	1.53	1.48	1.5	1.5
**TG 16:0_16:1_18:4**	1.59	1.44	1.4	1.37	1.31	1.34	1.33	1.33
**TG 14:0_16:0_18:4**	1.58	1.4	1.41	1.38	1.28	1.25	1.25	1.24
**HexCer 18:1;O2/24:1**	1.58	1.4	1.34	1.29	1.21	1.21	1.21	1.21
**TG 14:0_16:1_20:5**	1.56	1.38	1.39	1.35	1.26	1.26	1.25	1.25
**Cer 18:1;O2/24:1**	1.55	1.51	1.42	1.37	1.28	1.23	1.23	1.23
**TG 16:0_18:0_20:5**	1.55	1.38	1.39	1.35	1.27	1.23	1.22	1.22
**TG 18:0_20:5_22:6**	1.55	1.37	1.38	1.33	1.27	1.25	1.25	1.24
**DG 14:0_16:0**	1.54	1.49	1.41	1.37	1.33	1.38	1.37	1.38
**PC O-36:6**	1.54	1.38	1.29	1.25	1.5	1.48	1.47	1.46
**TG 14:0_18:4_20:5**	1.53	1.36	1.4	1.35	1.26	1.23	1.22	1.22
**TG 14:0_16:1_22:6**	1.52	1.35	1.42	1.38	1.28	1.26	1.26	1.25
**DG 16:0_16:1.1**	1.48	1.4	1.34	1.3	1.24	1.4	1.39	1.39
**TG 14:0_16:2_20:5**	1.47	1.31	1.34	1.31	1.22	1.26	1.26	1.25
**PI 38:4**	1.44	1.33	1.25	1.23	1.41	1.36	1.36	1.36
**TG 16:0_16:0_18:4**	1.42	1.31	1.32	1.3	1.24	1.2	1.19	1.19
**PE 40:8**	1.4	1.25	1.38	1.34	1.52	1.46	1.46	1.46
**TG 16:0_16:0_20:4**	1.4	1.36	1.33	1.31	1.24	1.19	1.19	1.19
**Cer 16:1;O2/16:0**	1.38	1.23	1.21	1.18	1.16	1.12	1.12	1.12
**DG 14:0_16:1**	1.37	1.51	1.59	1.59	1.82	1.87	1.91	1.92
**TG 18:0_22:6_22:6**	1.36	1.21	1.24	1.2	1.16	1.16	1.16	1.16
**PE 42:8.1**	1.36	1.23	1.26	1.22	1.14	1.1	1.09	1.09
**DG 18:0/18:0**	1.35	1.21	1.13	1.17	1.09	1.05	1.04	1.05
**DG 16:1_18:3**	1.33	1.37	1.31	1.27	1.18	1.14	1.15	1.14

**TG 14:0_18:4_22:6**	1.32	1.18	1.29	1.25	1.17	1.12	1.12	1.11
**TG 16:1_16:2_18:1**	1.29	1.22	1.2	1.18	1.28	1.25	1.24	1.24
**TG 16:0_20:1_20:5**	1.29	1.14	1.08	1.08	1.02	1.0	0.99	0.99
**DG 16:0_16:1**	1.27	1.58	1.48	1.44	1.33	1.28	1.27	1.27
**TG 16:0_16:0_22:6**	1.24	1.2	1.23	1.19	1.11	1.11	1.1	1.1
**PG 16:0_20:5**	1.21	1.08	1.31	1.28	1.4	1.34	1.34	1.34
**DG 18:3_22:5**	1.2	1.09	1.06	1.07	1.0	1.01	1.0	1.0
**DG 18:0_22:1.1**	1.2	1.13	1.06	1.06	1.04	1.0	1.0	1.0
**TG 16:0_20:1_22:6**	1.19	1.05	1.03	1.03	0.97	0.93	0.93	0.93
**DG O-40:8**	1.17	1.16	1.21	1.17	1.16	1.18	1.19	1.19
**LPE 20:1**	1.16	1.04	1.26	1.25	1.39	1.33	1.33	1.32
**PE 40:8.2**	1.16	1.09	1.27	1.23	1.14	1.11	1.1	1.11
**DG 16:2_22:6**	1.14	1.11	1.17	1.14	1.1	1.06	1.06	1.05
**TG 16:0_20:5_22:1**	1.13	1.01	0.97	0.97	0.91	0.89	0.88	0.88
**TG 18:1_18:4_20:5**	1.13	1.02	1.0	0.98	0.91	0.9	0.89	0.89
**LPC 18:0**	1.11	1.01	0.95	0.96	0.97	1.07	1.07	1.07
**PG 16:1_22:6**	1.11	1.0	1.15	1.11	1.22	1.18	1.17	1.17
**Cer 18:3;O2/24:1;O**	1.11	1.0	1.02	0.99	0.96	1.06	1.07	1.07
**Cer 18:0;O2/24:1**	1.1	1.05	1.07	1.03	1.02	1.03	1.03	1.03
**PC 36:5**	1.09	0.97	0.94	0.95	1.19	1.15	1.15	1.15
**PE O-38:6**	1.07	0.97	1.14	1.15	1.25	1.2	1.19	1.2
**TG 14:0_20:5_22:6**	1.07	0.99	1.11	1.08	1.02	0.98	0.97	0.97
**DG 16:1_18:1**	1.05	1.25	1.18	1.17	1.09	1.05	1.04	1.04
**TG 16:1_18:1_20:5**	1.05	0.93	0.94	0.92	0.98	0.98	0.98	0.98
**TG 16:0_18:1_22:6**	1.04	1.18	1.22	1.18	1.1	1.05	1.04	1.05
**TG 16:1_16:4_20:5**	1.03	0.93	1.11	1.08	1.01	1.05	1.05	1.05
**PC O-42:7**	1.0	1.02	1.0	0.98	1.05	1.01	1.0	1.01
